# Expectation Violation Influences Neural Responses to the Accessibility of Cognitions Related to Suicide and Life: A Simultaneous EEG-fNIRS Study

**DOI:** 10.3390/brainsci16040367

**Published:** 2026-03-28

**Authors:** Bo Liu, Yuntena Wu, Tonglin Jin, Zeyu Lei

**Affiliations:** 1 School of Psychology, Inner Mongolia Normal University, Hohhot 010022, China; liubopsy@163.com (B.L.); psyjin@sina.com (T.J.); leizeyu_psy@163.com (Z.L.); 2 Mental Health Education Research and Service Center, Key Research Base of Humanities and Social Science in Inner Mongolia Colleges and Universities, Hohhot 010022, China

**Keywords:** suicide, accessibility of suicidal cognitions, expectation violation, EEG, FNIRS

## Abstract

Background/Objectives: Increased accessibility of suicidal cognitions reflects the cognitive processes underlying the acquisition of suicidal thoughts. Previous research shows that expectation violation reduces the accessibility of life cognitions rather than increasing that of suicidal cognitions, but this may be due to a slowing effect masking an increase in suicidal cognitions. Methods: Beyond the reaction time task, the present study used simultaneous EEG-fNIRS to reveal how expectation violation differentially affects the accessibility of suicidal and life cognitions. In a trial-by-trial cognitive task, participants read sentences that were either semantically consistent (expectation confirmation) or anomalous (expectation violation), followed by a semantic judgment on suicide-related, neutral, and life-related words. Response times for each word type served as a measure of cognitive accessibility for that category. Results: Compared to expectation confirmation, expectation violation reduced the cognitive accessibility of life rather than increasing that of suicide in the reaction time task. However, in neural responses, it led to reduced N1 amplitude, increased P2 amplitude for suicide-related information, and greater hemodynamic response in the left frontopolar region. Conclusions: Expectation violation triggered distinct neural responses to suicidal information, reflecting an attentional bias that may explain how suicidal thoughts emerge within normative cognition.

## 1. Introduction

The accessibility of suicidal cognitions refers to the ease with which suicide-related thoughts come to mind and is considered the starting point of the entire suicidal process [1]. Although it is part of normal cognitive functioning, its frequent entry into awareness may develop into explicit suicidal thoughts or behaviors [1]. In basic psychological science, investigating the accessibility of suicidal cognitions helps advance our understanding of suicide [2]. Furthermore, laboratory-based causal investigations into the necessary conditions for suicide will also benefit suicide prevention efforts [3,4].

Previous research has suggested that the failure to meet expected standards in reality serves as a necessary condition for heightened accessibility of suicidal cognitions, primarily because it triggers the motivation to escape aversive self-awareness [1,5,6,7]. From the perspective of Terror Management Theory (TMT), both reality-expectation discrepancies that undermine self-esteem and threats to the cultural worldview can increase the accessibility of death cognitions [8,9,10]. Given the semantic association between suicide and death, a threatened cultural worldview will increase the accessibility of death cognitions and may spread such activation to the accessibility of suicidal cognitions [5]. Interestingly, a recent study situated threats to the cultural worldview within the broader framework of expectation violation, finding that expectation violation reduced the accessibility of life cognitions rather than increasing that of suicidal cognitions [11]. In this study, after reading content that violated both evolutionary perspectives and linguistic logic, participants performed a word semantic categorization task. The speed of recognizing suicidal words and life words respectively represented the accessibility of suicidal and life cognitions. Expectation violation induces a motor slowing effect by recruiting a global inhibitory neural network in the brain [12,13,14]. One possible explanation for why participants showed slower response times to life words, not faster responses to suicidal words, is that the general slowing induced by expectation violation may have masked a genuinely faster recognition speed for suicidal words. How the accessibility of suicidal and life cognitions changes following expectation violation remains unclear. In clinical populations, higher accessibility of suicidal cognitions is rooted in sustained attentional engagement with suicide-related information [15], which may also serve as the cognitive basis for how expectation violation influences the accessibility of suicidal and life cognitions. If expectation violation preferentially captures attention toward suicidal words, this would indicate that expectation violation indeed enhances the accessibility of suicidal cognitions, rather than merely reducing the accessibility of life cognitions. Given that reaction times alone cannot readily dissociate the motor slowing effect caused by expectation violation, and attentional engagement with information about suicide or life is a transient process, the present study employed electroencephalography (EEG), which offer high temporal resolution, to capture this rapid process. In the broader context of neural responses reflecting increased accessibility of suicidal cognitions following expectation violation, EEG directly represents neuronal firing, whereas functional near-infrared spectroscopy (fNIRS) reflects local hemodynamic changes resulting from metabolic demands after neuronal activity. To further investigate the neurovascular coupling underlying changes in the accessibility of suicidal and life cognitions [16,17], fNIRS was simultaneously recorded. fNIRS offers advantages such as low cost, portability, and relative robustness to motion artifacts, making its findings more readily applicable to clinical patients in naturalistic settings [18]. Both EEG and fNIRS have been widely used in studies of clinical patients with suicidality.

A substantial body of research using EEG has identified distinctive neural markers in clinical populations with suicidality. Abnormal N1 and P2 amplitudes during cognitive tasks in individuals with suicidal ideation or attempts reflect impairments in early attentional and perceptual processing [19]. During semantic judgments of death-related information, more negative N1 amplitudes (parietal and occipital regions) were associated with weaker self-death associations in adolescents, suggesting better top-down attentional control over death-related information [20]. Adolescents with suicidal thoughts or behaviors showed no difference in P2 amplitudes evoked by death words and life words, whereas healthy adolescents exhibited higher P2 amplitudes for life words than for death words [20]. Although this result did not directly show that suicidal words elicited significantly higher P2 amplitudes than life words in adolescents with suicidality, it suggests an attentional bias toward suicidal information in this group. Indeed, adolescents with suicide attempts elicited higher P2 amplitudes when viewing negative words compared to those with suicidal ideation only [21].

Studies using fNIRS to investigate neural features in clinical suicidal populations have primarily focused on prefrontal hemodynamic changes during cognitive tasks, with some studies, including those employing the Verbal Fluency Task, also examining temporal lobe activity [18]. Individuals with suicidality often fail to adequately recruit prefrontal regions to support optimal task performance. Compared to depressed patients without suicidal ideation, those with suicidal ideation show reduced activation in the dorsolateral prefrontal cortex (DLPFC), ventrolateral prefrontal cortex (VLPFC), and frontopolar cortex (FPC) during cognitive tasks [22,23], with this hypoactivation being associated with greater suicidal ideation [24]. Similarly, reduced prefrontal activation during cognitive tasks has been observed in comparisons between schizophrenia patients with and without suicide attempts [25]. However, direct fNIRS responses to suicidal stimuli in clinical suicidal populations remain unexplored. Furthermore, fNIRS indicators reflecting direct responses to suicidal information in healthy individuals have yet to be identified.

The primary aim of the present study is to utilize simultaneous EEG and fNIRS recordings to clarify the differential effects of expectation violation on the accessibility of suicidal and life cognitions. Drawing on previous research [11], expectation violation was induced by reading semantically illogical sentences, while the accessibility of suicidal and life cognitions was assessed by measuring recognition speeds for different word types. Consistent with previous findings [11], the present study hypothesized that expectation violation would reduce the accessibility of life cognitions in the reaction time task. Given that the slowing effect induced by expectation violation may mask an increase in the accessibility of suicidal cognitions, and that previous studies have observed neural responses to death-related information [20] and other cognitive tasks [18,19,21] in clinical patients, the present study further hypothesized that expectation violation would lead to decreased N1 amplitude, increased P2 amplitude in the parietal and occipital lobes, and reduced hemodynamic responses in the prefrontal cortex in response to suicide-related information. This study offers direct biological insight into how expectation violation influences the accessibility of suicidal and life cognitions. More broadly, it provides a neural interpretation of how threatened cultural worldview in TMT increases the accessibility of death cognitions—given the semantic association between death and suicide—and how healthy individuals acquire suicidal cognitions.

## 2. Materials and Methods

### 2.1. Participants

Based on the effect size (*f* = 0.22) reported in previous relevant research [6], parameters were set in G*Power 3.1.9.7 as *f* = 0.22 and α = 0.05, indicating that a sample of 24 participants would achieve a statistical power of 0.8. To maintain statistical power in case of potential data loss, 40 university students (24 women) were recruited through social media platforms. The mean age was 21.43 years (*SD* = 1.85). All participants were right-handed, had normal or corrected-to-normal vision, self-reported no history of psychiatric or neurological disorders, and had not taken any neuroactive medication within the past month. Each participant provided written informed consent and received monetary compensation upon completion of the experiment. The study was approved by the Institutional Research Ethics Committee (XL24042401).

### 2.2. Materials

A total of 120 sentences and 60 words were sourced from a previous study [11]. The sentence set included 60 semantically logical sentences (expectation confirmation) and 60 semantically illogical sentences (expectation violation), each composed of 11 Chinese characters. Compared to the logical sentences, the illogical sentences differed only in the final word (two characters). For example, a sentence under the expectation confirmation condition was “The children like to go outside to play”, while under the expectation violation condition it was “The children like to go outside to melt”. When participants read “The children like to go outside to”, they expected the sentence to end with “play”, and the word “melt” violated that expectation.

The 60 words consisted of 20 suicidal words (e.g., Hang oneself, Drink poison), 20 neutral words (e.g., Window, Book), and 20 life words (e.g., Survival, Breathing). Each word was composed of two Chinese characters and appeared once under both the expectation confirmation and expectation violation conditions. All words are available at the URL: https://www.frontiersin.org/articles/10.3389/fpsyg.2025.1680869/full#supplementary-material (accessed on 25 March 2026).

### 2.3. Design and Procedure

A 2 (Expectation condition: expectation confirmation, expectation violation) × 3 (Word type: suicide, neutral, life) within-subjects design was implemented, with both expectation condition and word type serving as within-subject variables.

**Figure 1:**
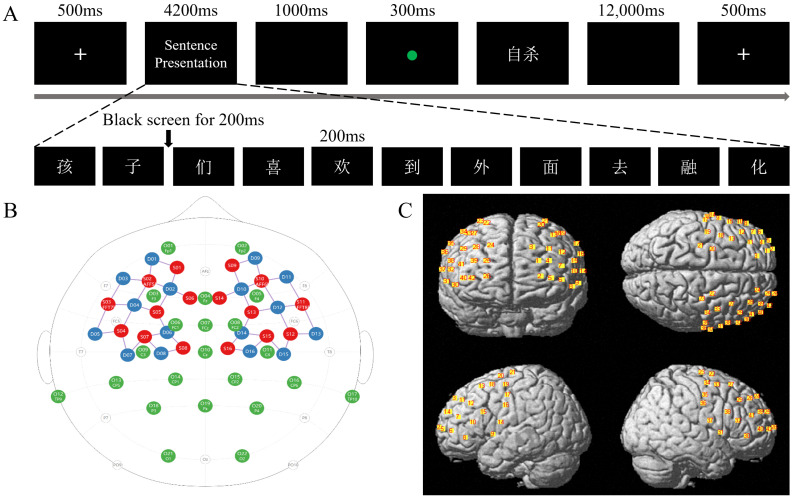
Schematic diagram of the experimental procedure (**A**) and neurosignal acquisition (**B**,**C**). In (**A**), the sentence presentation stage shows a sentence under the expectation violation condition: “The children like to go outside to melt.” The word judgment following the green dot presents the meaning of the word as “suicide”. (**B**) 2D layout of the recording positions: green denotes EEG electrode locations, red indicates fNIRS source positions, and blue marks fNIRS detector positions. (**C**) 3D visualization of fNIRS channel locations; red numbers within yellow rectangles indicate channel numbers.

The experiment was run in E-Prime 3.0. All participants completed 8 practice trials to familiarize themselves with the procedure. The formal experiment consisted of 120 trials, evenly divided into 4 blocks. A 30 s break was provided between blocks for participants to rest briefly. In each trial (Figure 1A), a fixation cross first appeared to alert participants that a sentence would be presented at the center of the screen. The sentences are presented as individual characters, with a 200 ms black screen interval between each character so that participants can clearly see the changes in the center of the screen. To ensure participants processed the meaning of the sentence, they were required to read each character aloud as it appeared. Following the sentence, a black screen appeared, followed by a green dot (allowing participants to clearly distinguish the sentence-reading phase from the word-judgment phase), indicating that a word would soon be presented. This word could be a suicide-related word, a neutral word, or a life-related word. Once the word appeared, participants had to press a key as quickly and accurately as possible to judge whether it was a suicidal word, a neutral word, or a life word. The word disappeared immediately after the keypress. The mapping of the three word types to response keys was counterbalanced across participants using a Latin-square design. The word presented after the green dot (lexical judgment phase) and the final word of the sentence following the fixation point (expectation violation induction phase) were unrelated. When reading sentences that violate linguistic logic, the semantic meaning of the final word deviates from the participants’ expected meaning. If the mental representation primed by this expectation violation is similar to the semantic representation of a particular word type in the subsequent lexical judgment task, then faster responses to that word type should be observed. Conversely, slower responses to a given word type indicate that the expectation violation primed a mental representation opposite to the semantic representation of that word type. All sentences and words were presented in random order. To allow hemodynamic activity to return to baseline, each trial ended with a 12 s black screen.

### 2.4. Acquisition of EEG and fNIRS Signals

EEG signals were recorded using 22 Ag/AgCl active electrodes (actiCHamp, Brain Products, Gilching, Germany) placed according to the 10–20 system (Figure 1B). The online reference was set to FCz, with a sampling rate of 1000 Hz. A separate electrode connected to AFz served as the ground. Prior to data acquisition, the impedance at all electrode sites was kept below 10 kΩ.

FNIRS signals were acquired using a continuous-wave system (NIRScout, NIRX, Berlin, Germany) at a sampling rate of 3.91 Hz. Sixteen light sources (emitting infrared light at 760 nm and 850 nm) and sixteen detectors formed 42 channels (Figure 1B), covering the entire prefrontal cortex and motor cortex (Figure 1C). The distance between each source and detector was fixed at 3 cm using rigid optode holders. NIRS-SPM [26] was used to obtain the MNI coordinates and Brodmann area (BA) coverage for each channel. The positional information of all channels is presented in Table 1.

### 2.5. Data Analysis

#### 2.5.1. Reaction Time

With reference to prior relevant studies [11,15], trials with incorrect responses and those with reaction times exceeding ±3 *SD*s from the overall mean were excluded. Furthermore, trials shorter than 100 ms or longer than 3000 ms were removed as they likely reflected anticipatory responses or attentional shifts. After excluding 97 error trials and 103 trials beyond ±3 *SD*s, no trials remained with reaction times below 100 ms or above 3000 ms. A total of 4600 trials were retained, resulting in an exclusion rate of 0.04%. Linear mixed-effects models (LMM) were fitted using the lme4 package in R [27]. Expectation condition and word type were included as fixed factors, and participant as well as word were included as random effects. Significant interactions are reported, and simple effects analyses [28] were conducted to further examine significant interactions, with multiple comparisons corrected using the Bonferroni method.

#### 2.5.2. EEG and fNIRS

EEG data were preprocessed using EEGLAB v2024.0 [29]. During offline analysis, the data were re-referenced to the average of the bilateral mastoids (TP9, TP10) and band-pass filtered at 0.01–30 Hz [30]. To improve processing speed, the sampling rate was downsampled to 500 Hz. Independent component analysis (ICA) in EEGLAB was applied to remove ocular and cardiac artifacts. To correct for motion artifacts, segments exceeding ±100 μV were rejected. The data were epoched from 200 ms before to 800 ms after stimulus onset, with the 200 ms pre-stimulus interval serving as the baseline for baseline correction. Based on previous studies [20,31] and visual inspection of the grand-average waveform, six electrode pools were defined. For the frontal (F3, F4, Fz), frontal/central (FC1, FC2, FCz), central (C3, C4, Cz), central/parietal (CP1, CP2, CP5, CP6), and parietal (P3, P4, Pz) regions, the mean amplitude in the 155–300 ms (P2) time window was computed. We also calculated the mean amplitude in the 150–210 ms (N1) and 228–284 ms (P2) time windows for the occipital region (O1, O2). Within each electrode pool, trials with incorrect responses and those exceeding ±3 *SD*s from the overall mean amplitude were excluded. Subsequently, LMMs were fitted for all trials in each electrode pool. Significant interaction effects were reported and followed up with simple effects analyses, with multiple comparisons corrected using the Bonferroni method.

FNIRS data from three participants were excluded due to a power failure of the recording device. The raw light intensity data were band-pass filtered between 0.01 and 0.2 Hz in nirsLAB_v201904, and spike correction was applied to values exceeding 5 *SD*s of the local mean [30]. Based on the modified Beer–Lambert law [32], light intensity was converted into changes in hemoglobin concentration. Given its higher sensitivity in detecting neural activity [30], the present study focused only on oxygenated hemoglobin (HbO). A 12 s time window following stimulus onset was defined, and all HbO values within this window were extracted for each trial. Eight regions of interest (ROIs) were defined according to the BA locations of the channels: bilateral frontopolar cortex, bilateral DLPFC, bilateral VLPFC, and bilateral motor cortex (see Table 1). The HbO for each ROI was averaged across all channels within that ROI. A general linear model (GLM) convolved with a hemodynamic response function was used to estimate trial-wise *β* values, which served as the indicator of brain activation [30]. Within each ROI, trials with incorrect responses (same as in the reaction-time task) and those exceeding ±3 *SD*s from the overall mean β were excluded. Subsequently, LMMs were fitted for all trials in each ROI. Significant interaction effects were reported and followed up with simple effects analyses, with multiple comparisons corrected using the Bonferroni method.

## 3. Results

### 3.1. Reaction Time

The LMM results are presented in Table 2. Compared to expectation confirmation, expectation violation led to a significant slowing of responses (expectation confirmation: *M* = 768.09, *SD* = 234.30; expectation violation: *M* = 787.93, *SD* = 243.32). The main effect of word type indicated that the cognitive accessibility of life (*M* = 836.94, *SD* = 256.12) was significantly lower than that of suicide (*M* = 752.35, *SD* = 234.05) and neutral (*M* = 748.56, *SD* = 216.57). Furthermore, the reduced cognitive accessibility of life was modulated by expectation condition. A significant interaction was found only between the cognitive accessibility of suicide and that of life when comparing expectation confirmation and expectation violation (expectation confirmation—suicidal words: *M* = 752.76, *SD* = 231.08; expectation confirmation—life words: *M* = 811.77, *SD* = 250.22; expectation violation—suicidal words: *M* = 751.93, *SD* = 237.17; expectation violation—life words: *M* = 862.92, *SD* = 259.70). Simple effects analysis indicated no significant difference in the cognitive accessibility of suicide between expectation confirmation and expectation violation (*β* = −2.47, *SE* = 9.31, *z* = −0.27, *p* = 0.791). However, the cognitive accessibility of life following expectation violation was significantly lower than under expectation confirmation (*β* = −53.49, *SE* = 9.63, *z* = −5.56, *p* < 0.001).

### 3.2. EEG

#### 3.2.1. N1 Component

**Figure 2:**
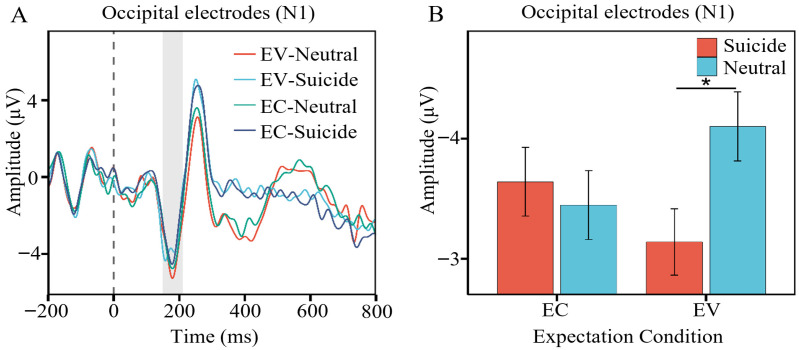
Significant interaction between expectation condition and word type on the N1 component. (**A**) Waveforms for suicidal words and neutral words under the two expectation conditions. (**B**) Mean amplitudes for suicidal words and neutral words under the two expectation conditions. The error bars represent the standard error, EC = Expectation Confirmation, EV = Expectation Violation. *, *p* < 0.05.

A significant interaction between suicidal words and neutral words in the comparison of expectation confirmation and expectation violation was observed in the occipital region (Figure 2), *β* = 1.15, *SE* = 0.55, *z* = 2.10, *p* = 0.035. Under expectation confirmation, there was no significant difference between suicidal words and neutral words (*β* = 0.19, *SE* = 0.39, *z* = 0.49, *p* > 0.999). Under expectation violation, however, suicidal words elicited a significantly smaller N1 amplitude than neutral words (*β* = 0.96, *SE* = 0.39, *z* = 2.49, *p* = 0.039).

#### 3.2.2. P2 Component

**Figure 3:**
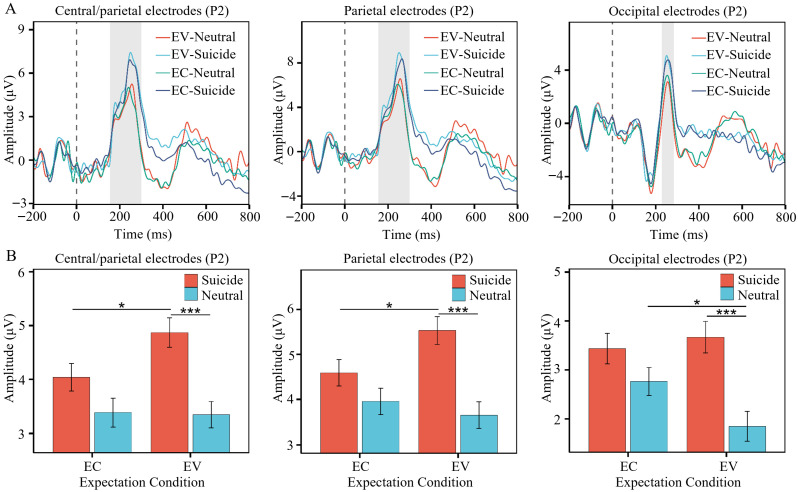
Significant interaction between expectation condition and word type on the P2 component. (**A**) Waveforms for suicidal words and neutral words under the two expectation conditions. (**B**) Mean amplitudes for suicidal words and neutral words under the two expectation conditions. The error bars represent the standard error, EC = Expectation Confirmation, EV = Expectation Violation. *, *p* < 0.05; ***, *p* < 0.001.

A significant interaction between suicidal words and neutral words in the comparison of expectation confirmation and expectation violation was observed in the central/parietal (*β* = 1.00, *SE* = 0.50, *z* = 2.01, *p* = 0.044), parietal (*β* = 1.22, *SE* = 0.56, *z* = 2.17, *p* = 0.029), and occipital (*β* = 1.13, *SE* = 0.57, *z* = 2.00, *p* = 0.045) regions (Figure 3). When word type was held constant, neutral words showed no significant difference between expectation confirmation and expectation violation in the central/parietal (*β* = 0.13, *SE* = 0.35, *z* = 0.36, *p* = 0.723) and parietal (*β* = 0.30, *SE* = 0.40, *z* = 0.76, *p* = 0.446) regions. In contrast, suicidal words elicited significantly larger amplitudes under expectation violation than under expectation confirmation in these regions (central/parietal: *β* = 0.88, *SE* = 0.35, *z* = 2.49, *p* = 0.0129; parietal: *β* = 0.92, *SE* = 0.40, *z* = 2.31, *p* = 0.021). The occipital region, however, showed the opposite pattern: no significant difference for suicidal words between expectation conditions (*β* = 0.24, *SE* = 0.40, *z* = 0.61, *p* = 0.544), while neutral words elicited significantly smaller amplitudes under expectation violation than under expectation confirmation (*β* = 0.89, *SE* = 0.40, *z* = 2.23, *p* = 0.026).

When expectation condition was held constant, there was no significant difference between suicidal and neutral words under expectation confirmation in any of the three regions (central/parietal: *β* = 0.59, *SE* = 0.35, *z* = 1.69, *p* = 0.274; parietal: *β* = 0.60, *SE* = 0.40, *z* = 1.50, *p* = 0.405; occipital: *β* = 0.68, *SE* = 0.40, *z* = 1.70, *p* = 0.265). Under expectation violation, however, suicidal words elicited significantly larger mean amplitudes than neutral words across all three regions (central/parietal: *β* = 1.59, *SE* = 0.35, *z* = 4.53, *p* < 0.001; parietal: *β* = 1.82, *SE* = 0.40, *z* = 4.57, *p* < 0.001; occipital: *β* = 1.82, *SE* = 0.40, *z* = 4.55, *p* < 0.001).

### 3.3. FNIRS

**Figure 4:**
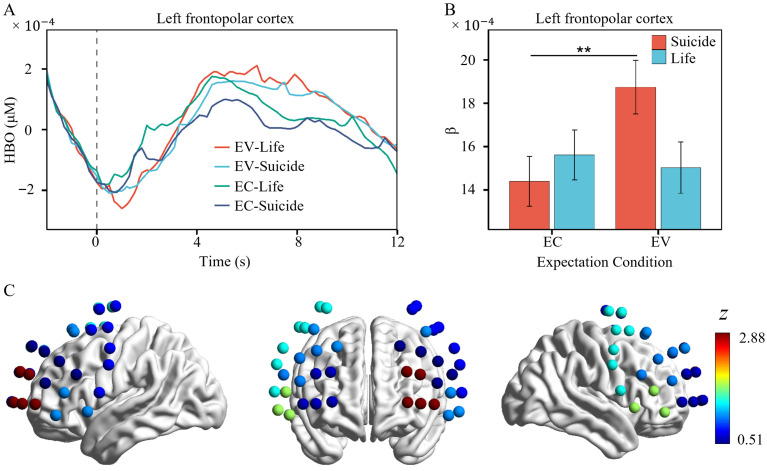
Significant interaction between expectation condition and word type in HBO in the left FPC. (**A**) HBO changes for suicidal words and life words under the two expectation conditions. (**B**) β values for suicidal words and life words under the two expectation conditions. (**C**) Activation topography for the comparison between suicidal words under expectation violation and expectation confirmation. The error bars represent the standard error, EC = Expectation Confirmation, EV = Expectation Violation. **, *p* < 0.01.

A significant interaction between suicidal words and life words in the comparison of expectation confirmation and expectation violation was found only in the left FPC (Figure 4), *β* = 0.0004431, *SE* = 0.0002139, *z* = 2.07, *p* = 0.038. Simple effects analysis revealed no significant difference in HbO for life words between expectation confirmation and expectation violation (*β* = 0.00199, *SE* = 0.0239, *z* = 0.08, *p* = 0.934). In contrast, activation for suicidal words was significantly higher under expectation violation than under expectation confirmation (*β* = 0.06728, *SE* = 0.0234, *z* = 2.88, *p* = 0.004).

### 3.4. Cross-Modal Correlations

Based on the above results, difference scores were computed for each participant between expectation violation and expectation confirmation for the mean reaction times to suicidal and life words, the mean N1 amplitude (occipital), the mean P2 amplitudes (central/parietal, parietal, occipital), and the mean β in the left FPC. These difference scores represent the increment in the accessibility of suicidal or life cognitions induced by expectation violation. All difference scores were converted to standardized z-scores, and Pearson correlation analyses were conducted. Across modalities, only one significant correlation was found: the increased activation for suicidal words under expectation violation in the left FPC (HBO) was positively correlated with the larger P2 amplitude in the occipital region (*r* = 0.36, *p* = 0.027).

## 4. Discussion

The present study characterized the neural responses of expectation violation affecting the accessibility of suicidal and life cognitions using EEG and fNIRS. Compared with expectation confirmation, expectation violation slowed responses to life words in the reaction time task, but elicited higher P2 amplitudes (central/parietal and parietal regions) for suicidal words in EEG and higher HBO (left FPC) in fNIRS.

TMT posits that, when confronted with death information, humans exhibit defensive responses aimed at upholding their cultural worldviews. Conversely, threats to cultural worldviews have been shown to increase the accessibility of death cognitions [9,10]. While death symbolizes the end of one’s physical and psychological existence, suicide represents an escape from physical and psychological suffering [33]. Given the shared connotations and semantic relatedness between suicide and death, threats to cultural worldviews may lead to a spreading activation from death information to suicide information [5]. Responses that violate one’s worldview occur within the broader framework of expectation violation [34]. Using traditional methods of threatening cultural worldviews (e.g., challenging one’s beliefs about evolution), previous research has revealed the flip side of expectation violation increasing the accessibility of suicide cognitions, namely a reduction in the accessibility of life cognitions [11]. Although the present study did not employ traditional worldview threat manipulations, it consistently observed that expectation violation reduced the accessibility of life cognitions rather than increased the accessibility of suicide cognitions, aligning with the findings of prior research [11]. This result does not rule out the possibility that the slowing effect induced by expectation violation may have masked faster responses to suicidal words. In line with earlier findings [12,13], the current study did observe a general slowing effect under expectation violation in the reaction time task. The slower responses to life words under expectation violation likely stem from this general slowing. Conversely, expectation violation might actually facilitate faster responses to suicidal words, but this facilitation could have been masked by the overall response slowing, preventing the observation of any difference in response speed to suicidal words between expectation confirmation and expectation violation. In contrast, neural responses to suicidal words were directly observed in EEG and fNIRS, showing clear differences between expectation confirmation and expectation violation.

Compared with neutral words, the smaller N1 amplitude elicited by death words in healthy populations suggests a death-related semantic processing stage that precedes emotional reactions, as it is directly linked to higher pessimism [35]. In clinical populations, the smaller occipital N1 amplitude for death words is associated with stronger implicit death-self associations, which may reflect a deficit in top-down attentional control in individuals with more severe suicidal symptoms [20]. Consistent with these findings, the present study observed that expectation violation led to a smaller N1 amplitude for suicidal words than for neutral words in the occipital region. One possible explanation is that expectation violation induces a state in which suicide-related information gains easier access to awareness, thereby making it more difficult for participants to exert attentional control over such information.

Suicidal information elicits larger P2 amplitudes in clinical suicidal populations, which has been interpreted as reflecting greater attentional bias and arousal toward suicide-related information in these individuals [20]. Similarly, larger P2 amplitudes in parietal and occipital regions have been observed in patients with major depressive disorder when responding to negative information, suggesting that depressed patients persistently allocate attentional control toward negative stimuli [36]. Consistent with these findings, the present study found that expectation violation led to larger P2 amplitudes for suicidal words compared to both expectation confirmation and to neutral words under expectation violation. Interestingly, the larger P2 amplitude for suicidal words than for neutral words in the occipital region under expectation violation was primarily due to a reduction in P2 amplitude for neutral words. One possible explanation is that during expectation violation, the occipital region allocated more attentional control to neutral words in the 150–210 ms window (N1), resulting in reduced attentional control to neutral words in the subsequent 228–284 ms window (P2).

fNIRS showed the same interaction pattern as reaction times in the comparison between suicidal and life words across the two expectation conditions. Inconsistent with the behavioral results, however, this interaction was driven by greater activation for suicidal words, not for life words, in the left FPC under expectation violation. Clinical suicidal populations typically exhibit deactivation in the FPC during cognitive tasks, which has been interpreted as reflecting prefrontal dysfunction in these individuals [24,37,38]. The observed PFC activation in the present study may stem from the use of a non-clinical sample without marked dysfunction, who performed a normative cognitive experimental task. A core function of the FPC is to serialize cognitive processing across multiple tasks: pending tasks are temporarily held in the PFC, and the FPC is prominently engaged when a pending task switches to become the currently processed one [39]. In the current reaction time task, participants were required not only to judge word meaning but also to maintain the response-key mappings for three word types. Activation in the left PFC may reflect increased difficulty in attentional control toward word meaning, necessitating greater cognitive resources for subsequent key-press responses. The positive correlation between higher left FPC activation elicited by expectation violation and larger P2 amplitude in the occipital region appears to support this view. Unfortunately, the anatomical location of the left PFC does not directly correspond to the occipital lobe, and the neurovascular coupling process underlying this correlation remains unclear.

Several limitations must be acknowledged. First, the present study did not dissociate the effects of word meaning and valence, which may affect the purity of the results. Although previous research has shown that the effect of expectation violation on the accessibility of suicide cognitions is independent of general negative cognitive accessibility [11], the stronger negative valence of suicide words suggests that using negative words as a control may not be optimal. Moreover, the categorization among three word types may introduce an additional motor decision-making process. Future studies using suicide pictures (with murder as a control) [40] and simpler word judgment tasks may improve the purity of the effect of expectation violation on the accessibility of suicide cognitions. Second, in accordance with previous research [18], fNIRS only covered the frontal lobe. The absence of significant EEG findings in the frontal lobe should be interpreted with caution. Although optical signals theoretically do not affect electrical signals [16], the possibility that current generated by the light source device during illumination may influence EEG signals cannot be ruled out. Future studies should include a separate whole-brain EEG analysis, expand fNIRS coverage to the temporal and parietal lobes, or integrate fMRI to provide a more comprehensive neural mapping. Third, the small-sample laboratory design may limit the generalizability of the findings. Future research should test the effects in populations beyond healthy college students, particularly in clinical patients. Fourth, the method used to induce expectation violation may limit ecological validity. While reading sentences that violate linguistic logic has been considered a minimal expectation violation manipulation in previous studies [11,41], real-world expectation violations typically involve clear valence. Future research should employ more ecologically valid methods to induce expectation violation, examine the differential effects of positive versus negative expectation violation, and dissociate the effects of expectation violation per se from those of valence. Fifth, given the possibility that additional assessments after each trial might interfere with fNIRS results, the present study did not include additional attention or emotion assessments. The interpretation of N1 and P2 amplitude changes as reflecting attentional bias is based on findings from previous studies [19]. Future studies should incorporate explicit attention and emotion assessment tasks to link neural responses to cognitive and emotional processes. Sixth, the lack of a direct association between reaction time and neural responses is regrettable, as it weakens the cognitive interpretability of the neural findings. Future research could employ more fine-grained cognitive tasks or introduce drift–diffusion modeling and eye tracking to clarify the cognitive processes underlying neural responses. Seventh, certain individual differences may have been overlooked. Previous studies have shown that clinical suicide symptoms [42] and locus of control [7] moderate the effect of negative expectation violation on the accessibility of suicide cognitions, and these factors may similarly modulate individuals’ responses to suicide stimuli following expectation violation. Incorporating additional moderators in future research may reveal important individual differences. Eighth, due to limitations in spatial registration, the simultaneous acquisition of EEG and fNIRS did not reach its full potential. Placing electrodes at the center of light emitters and detectors [43] may facilitate modeling both modalities into a unified pattern of neural response. Finally, the primary aim of the present study was to establish a necessary cognitive basis for the emergence of suicidal thoughts. To clarify this causal inference, future longitudinal studies in clinical patients are needed.

## 5. Conclusions

In summary, building on previous findings that expectation violation reduces the accessibility of life cognitions [11], the present study provides further neural insights. The slower responses to life words in the reaction-time task under expectation violation likely resulted from a general slowing that masked faster responses to suicidal words. Expectation violation led to lower N1 amplitude (occipital), higher P2 amplitude (central/parietal, parietal, occipital), and greater left PFC activation for suicidal words, but not for life words. These patterns suggest that expectation violation may enhance attentional bias toward suicide-related information.

## Figures and Tables

**Table 1:** Coordinates of all fNIRS channels.

ROI	Channel	BA	Brain Area Coverage	MNI		
				x	y	z
Left frontopolar cortex	1	10	0.77	−33.50	74.90	31.87
Left frontopolar cortex	2	10	1	−34.16	80.67	5.43
Left frontopolar cortex	4	10	0.81	−45.16	67.26	29.44
Left frontopolar cortex	5	10	1	−45.54	73.14	4.36
Left frontopolar cortex	6	10	0.72	−57.33	61.94	3.78
Left dorsolateral prefrontal cortex	3	9	0.51	−27.45	64.66	53.95
Left dorsolateral prefrontal cortex	7	46	0.76	−59.871	53.246	22.053
Left dorsolateral prefrontal cortex	11	9	0.63	−45.787	53.85	49.061
Left dorsolateral prefrontal cortex	12	9	0.61	−60.524	40.956	40.743
Left dorsolateral prefrontal cortex	15	9	0.57	−72.892	26.261	29.432
Left ventrolateral prefrontal cortex	8	47	0.70	−69.74	44.01	−5.752
Left ventrolateral prefrontal cortex	10	45	0.60	−71.574	36.802	13.685
Left superior temporal gyrus #	9	22	0.62	−78.798	16.558	−2.567
Left includes frontal eye fields #	13	8	0.66	−50.145	28.256	66.325
Left pre-motor and supplementary motor cortex	14	6	0.50	−81.152	6.245	13.772
Left pre-motor and supplementary motor cortex	16	6	0.78	−79.501	−1.83	37.786
Left pre-motor and supplementary motor cortex	17	6	0.72	−74.694	−2.92	52.036
Left pre-motor and supplementary motor cortex	18	6	0.92	−56.776	13.231	67.823
Left pre-motor and supplementary motor cortex	19	6	0.67	−61.102	−1.178	70.025
Left pre-motor and supplementary motor cortex	20	6	1	−38.309	6.192	85.636
Left pre-motor and supplementary motor cortex	21	6	0.95	−41.74	−7.888	88.352
Right frontopolar cortex	25	10	0.76	32.686	75.541	31.475
Right frontopolar cortex	26	10	1	31.441	82.027	5.911
Right frontopolar cortex	39	10	0.70	45.445	66.723	30.055
Right frontopolar cortex	40	10	0.69	56.612	62.741	4.051
Right frontopolar cortex	42	10	1	45.39	73.27	4.23
Right dorsolateral prefrontal cortex	28	9	0.52	44.812	54.123	49.962
Right dorsolateral prefrontal cortex	29	9	0.64	59.485	40.478	43.518
Right dorsolateral prefrontal cortex	38	9	0.69	73.48	25.07	29.142
Right dorsolateral prefrontal cortex	41	46	0.81	59.26	53.434	23.662
Right ventrolateral prefrontal cortex	30	47	0.62	68.41	46.6	−5.307
Right ventrolateral prefrontal cortex	32	45	0.61	71.65	36.449	14.326
Right includes frontal eye fields #	24	8	0.54	27.208	65.286	53.362
Right includes frontal eye fields #	27	8	0.57	47.273	29.649	68.338
Right superior temporal gyrus #	31	22	0.60	78.346	18.414	−1.154
Right pre-motor and supplementary motor cortex	22	6	1	36.156	6.763	86.608
Right pre-motor and supplementary motor cortex	23	6	0.97	41.605	−5.227	87.669
Right pre-motor and supplementary motor cortex	33	6	1	54.815	13.687	69.605
Right pre-motor and supplementary motor cortex	34	6	0.79	59.692	1.554	70.329
Right pre-motor and supplementary motor cortex	35	6	0.67	73.972	−0.795	52.16
Right pre-motor and supplementary motor cortex	36	6	0.67	79.448	−1.989	38.222
Right pre-motor and supplementary motor cortex	37	6	0.43	81.318	5.673	14.617

#. Channels that were excluded from being treated as ROIs because of constraints in their count.

**Table 2:** Results of the LMM for Reaction Time.

	*β*	*SE*	*t*	*p*	95%CI
Intercept	787.11	24.71	31.86	**<0.001**	[738.68, 835.54]
EC vs. EV	23.74	8.22	2.89	**0.005**	[7.64, 39.84]
Suicide vs. Neutral	−8.65	14.48	−0.60	0.553	[−37.03, 19.74]
Life vs. Neutral	−98.17	14.69	−6.68	**<0.001**	[−126.96, 69.38]
Suicide vs. Life	89.53	14.55	6.15	**<0.001**	[61.01, 118.04]
(EC vs. EV) × (Life vs. Neutral)	−38.23	22.82	−1.68	0.098	[−82.95, 6.49]
(EC vs. EV) × (Suicide vs. Neutral)	12.79	22.68	0.56	0.575	[−31.67, 57.25]
(EC vs. EV) × (Suicide vs. Life)	51.02	13.39	3.81	**<0.001**	[24.78, 77.25]

EC = Expectation Confirmation; EV = Expectation Violation; statistically significant effects are highlighted in bold.

## Data Availability

The raw data supporting the conclusions of this article will be made available by the authors, without undue reservation.
